# Corpus callosum morphology and relationships to illness phenotypes in individuals with anorexia nervosa

**DOI:** 10.1038/s41598-024-61841-6

**Published:** 2024-05-15

**Authors:** Jamie D. Feusner, Alicja Nowacka, Ronald Ly, Eileen Luders, Florian Kurth

**Affiliations:** 1 Centre for Addiction and Mental Health, 250 College St., Toronto, ON M5T 1R8 Canada; 2 Department of Psychiatry, University of Toronto, Toronto, Canada; 3 Department of Women’s and Children’s Health, Karolinska Hospital, Karolinska Institutet, Stockholm, Sweden; 4 Department of Psychiatry and Biobehavioral Sciences, University of California Los Angeles, Los Angeles, CA USA; 5 School of Psychology, University of Auckland, Auckland, New Zealand; 6 Department of Women’s and Children’s Health, Uppsala University, Uppsala, Sweden; 7 Laboratory of Neuro Imaging, School of Medicine, University of Southern California, Los Angeles, CA USA; 8 Departments of Neuroradiology and Radiology, Jena University Hospital, Jena, Germany

**Keywords:** Eating disorders, MRI, Morphometry, Shape concerns, Obsessive–compulsive symptoms, Biomarkers, Neuroscience, Psychology, Signs and symptoms

## Abstract

Anorexia nervosa is an often-severe psychiatric illness characterized by significantly low body weight, fear of gaining weight, and distorted body image. Multiple neuroimaging studies have shown abnormalities in cortical morphology, mostly associated with the starvation state. Investigations of white matter, while more limited in number, have suggested global and regional volume reductions, as well as abnormal diffusivity in multiple regions including the corpus callosum. Yet, no study has specifically examined thickness of the corpus callosum, a large white matter tract instrumental in the inter-hemispheric integration of sensory, motor, and cognitive information. We analyzed MRI data from 48 adolescents and adults with anorexia nervosa and 50 healthy controls, all girls/women, to compare corpus callosum thickness and examined relationships with body mass index (BMI), illness duration, and eating disorder symptoms (controlling for BMI). There were no significant group differences in corpus callosum thickness. In the anorexia nervosa group, severity of body shape concerns was significantly, positively correlated with callosal thickness in the rostrum, genu, rostral body, isthmus, and splenium. In addition, there were significant positive correlations between eating disorder-related obsessions and compulsions and thickness of the anterior midbody, rostral body, and splenium. There were no significant associations between callosal thickness and BMI or illness duration. In sum, those with AN with worse concerns about bodily appearance and worse eating disorder-related obsessive thought patterns and compulsive behaviours have regionally thicker corpus callosum, independent of current weight status. These findings provide important neurobiological links to key, specific eating disorder behavioural phenotypes.

## Introduction

Anorexia nervosa (AN) is an eating disorder characterized by a morbid fear of gaining weight and being fat, restriction of caloric intake and/or engaging in excessive exercise to the point of having a very low body weight, and disturbances in body image^1^. AN can take a particularly ominous course and severely affect individuals’ function and well-being, and disrupt their family’s lives. AN typically onsets in early adolescence and, in many, can continue into adulthood. Its crude mortality rate of 5.6% per decade^2^ is second only to opiate use disorder as the most life-threatening of psychiatric illnesses.

There are few effective treatments for AN, which may be due to an incomplete understanding of its neurobiology. The severe malnutrition state that individuals with AN experience can result in prominent brain structural changes. Multiple structural neuroimaging studies in AN have shown abnormalities in cortical morphology, including reductions in cortical thickness and subcortical volumes; these likely reflect atrophy from malnutrition and the starvation state since they tend to reverse when weight is restored^3,4^.

Overall, there have been fewer investigations of brain white matter, compared with gray matter, abnormalities in AN. One of the known effects of starvation is that it reduces white matter myelination. For example, a reduction in myelination in starvation states has been demonstrated experimentally in rodents and has been shown to be only partially restored by nutritional rehabilitation^5–7^. Reduction in myelination has also been demonstrated in malnutrition and starvation states in humans^8^, including in a small study in underweight adolescent females^9^.

Several studies in adolescents with AN have examined white matter volumes, although there have been inconsistent findings. In those with acute, underweight AN, two studies found reduced global white matter volume in adolescents with AN compared with healthy controls^10,11^ while another found no abnormalities^12^. In terms of regional abnormalities, one study found reductions in bilateral superior longitudinal fasciculi, superior thalamic radiation, corona radiata, fornix, pons, and medulla^13^. Also inconsistent across studies is whether reductions in global or regional white matter volume are solely a function of the underweight state. Two studies in weight-recovered adolescents with AN found no global or regional white matter volume abnormalities^14,15^, while one study found reduced global white matter volume compared with healthy controls^10^ and another found regional reductions in the pons^13^.

Neuroimaging studies of white matter in AN using diffusion tensor imaging (DTI) have revealed widespread abnormal diffusion patterns. Briefly, DTI is a specialised MRI technique that analyzes the degree and direction of diffusion of water molecules in brain tissue, providing estimates of microstructural properties. One way in which diffusion patterns can be characterized in white matter tracts is by fractional anisotropy (FA); lower FA could indicate less organized or potentially disrupted white matter tracts. A meta-analysis of DTI studies in AN found evidence, in the 10 included studies, of reduced FA in the left thalamus and left corona radiata^16^. Another quantitative meta-analysis, including 13 studies), found lower FA in AN compared with controls in the posterior corpus callosum (CC) body, the left superior longitudinal fasciculus II, the left precentral gyrus, and higher FA in the right cortico-spinal projections and lingual gyrus^17^. A meta-analysis of DTI tract-based spatial statistics studies found evidence of lower FA in the CC and the cingulum, but no association of diffusion measures with age or BMI^18^. The CC regions identified in this meta-analysis were primarily in the CC body and contained fibres connecting right and left prefrontal cortex and supplementary motor area (SMA). The authors posited that reduced FA in the body of the CC could result in reduced information transfer, which in turn could affect cognitive and affective aspects of body image distortion. A more recent study (not covered in these meta-analyses) found reduced FA in the CC body in AN and recovered AN, and reduced mean diffusivity (MD) in the posterior thalamic radiation in acute AN, compared with healthy controls^19^. Another more recent study found reduced white matter tract volume in AN (but not recovered AN) compared with healthy controls in anterior and mid-anterior portions of the CC^20^. Other recent studies, while not revealing abnormal diffusion measures in the CC, found increased FA in occipital and parietal regions^21^, and reduced structural connectivity (based on streamlines) within subcortical networks along with greater frontal cortical connectivity^22^.

These studies suggest that white matter microstructure abnormalities—as indexed by reduced FA and reduced tract volume—may be present in the CC (amongst other areas) in those with AN. The CC is a large white matter tract that connects the right and left hemispheres and facilitates interhemispheric transfer and integration of sensory, motor, and cognitive information^23^. The CC can be geometrically divided into three major regions: the genu + rostrum, connecting frontal and premotor regions of the two hemispheres; the body, which connects motor, somatosensory, and parietal areas; and the splenium + isthmus, which connects occipital and temporal cortices^24^. The CC is involved in homotopic and heterotopic cortical inter-hemispheric synchronizations. Homotopic synchronization refers to brain regions in one hemisphere synchronizing their activity with their counterparts in the opposite hemisphere, and thus being responsible for coordinating and integrating information between mirrored areas of the brain. In contrast, heterotopic inter-hemispheric synchronization refers to synchronization of brain regions that do not have direct correspondence across hemispheres, and thus relay information between non-matching brain regions. Both synchronizations play a critical part in coordinated action and perception^25,26^.

AN, like most psychiatric disorders, involves complex and broad pathophysiology including dysfunction in emotion regulation, habitual behaviours, cognitive control, social cognition, visual perception, and visuospatial functioning^27–29^. A meta-analysis of neuropsychological functioning studies in adults with AN found the largest effect sizes for impairments in visuospatial abilities, which were moderated by age (older performing worse) and BMI (lower BMI associated with worse performance)^30^. Body image distortion is a core characteristic, and a DSM-defining criterion, of AN^1^. Multiple studies have empirically demonstrated perceptual distortions for appearance in those with AN (reviewed in^31^). Given the role of the CC in homotopic and heterotopic hemispheric integration of information coming from parietal, occipital, and temporal regions involved in visuospatial functioning, somatosensory cortices involved in body perception, and prefrontal regions involved in higher cognitive functions such as self-appraisals, dysfunction may contribute to relevant symptomatology in AN.

Additional important, and common, phenomenological features in AN are eating disorder-related obsessive thoughts and ritualistic/compulsive behaviours^32^. Further, obsessive–compulsive disorder (OCD) comorbidity is high in those with AN^33,34^, and there is evidence of significant genetic correlations between AN and obsessive–compulsive disorder (OCD)^35^. Further, a study in individuals with OCD found thinner anterior and posterior CC regions and associations with visuospatial performance^36^ and another study in OCD found associations between severity of obsessions and compulsions and CC thickness^37^. However, no study has specifically examined CC morphology in adults or adolescents with AN nor relationships with body image disturbance, obsessions and compulsions, nor any other symptom domain.

The goal of this analysis was to characterize corpus callosum morphology in individuals with AN compared with healthy controls and to examine relationships with illness factors including weight/starvation-related effects (estimated by current body mass index—BMI), total illness duration, and the behavioural phenotypes of body shape concerns and eating disorder-related obsessions and compulsions. To achieve this, we analyzed data from T1-weighted MRI scans in adolescents and adults with AN and in healthy controls and measured the thickness of the CC at 100 equally spaced nodes^38^. This method has previously been used to identify abnormalities in several psychiatric disorders including OCD, schizophrenia, and autism spectrum disorder^36,39,40^.

We hypothesized that there would be abnormalities in CC thickness in those with AN compared with healthy controls. In addition, we hypothesized that CC thickness would be positively associated with BMI in those with AN, reflecting relationships with starvation and weight status. We also predicted that duration of illness would be inversely associated with thickness in the CC in those with AN who were acutely underweight and partially weight-restored, reflecting a cumulative impact of starvation-related and/or malnutrition effects on white matter. Further, we predicted in AN participants, controlling for BMI, significant associations between CC thickness and Eating Disorder Examination (EDE) shape concern subscale scores—indexing disturbances in body image, and Yale–Brown–Cornell Eating Disorders Scale scores—a measure of eating disorder-related obsessive thoughts and ritualistic/compulsive behaviours. We did not have specific directional predictions of associations with clinical variables since this was the first exploration of CC thickness in AN. Hypotheses were preregistered (AsPredicted #62141).

## Methods and materials

### Participants

We included participants’ data from two separate studies; study 1 in adults with AN and study 2 in adolescents with AN, each with their own cohorts of control participants. Detailed descriptions of the inclusion and exclusion criteria for both samples were as previously reported^41^. Recruitment of the adult sample was from local specialized treatment centres, online and community-based advertisements, and campus flyers at the University of California Los Angeles (UCLA). Recruitment of the adolescent sample was from the UCLA inpatient eating disorder unit and from local treatment centres; they were enrolled at the end of their treatment in these settings when they met each treatment centre’s individual criteria for transitioning to a lower level of care.

The study's protocols were approved by the UCLA Institutional Review Board, and all methods adhered to their guidelines and regulations. Written informed consent was obtained from all participants, while for the adolescent study, informed consents were acquired from parents and/or legal guardians and assents were obtained from the adolescents.

### Diagnostic and psychometric assessments

Licensed psychiatrists or psychologists experienced in working with individuals with AN conducted clinical evaluations and administered clinician-rated scales for all participants. The Mini-International Neuropsychiatric Interview (MINI v. 6.0 for adults^42^ and the MINI KID 7.0.2 for adolescents^43^) was used to screen for primary or comorbid diagnoses. The Eating Disorders Examination (EDE, version 16.0D) was used to determine total eating disorder symptom severity as well as shape concern subscale scores^44^. The severity of eating- and body/weight-related preoccupations and rituals was assessed using the Yale–Brown–Cornell Eating Disorder Scale (YBC-EDS)^45^. The Hamilton Anxiety Rating Scale (HAMA)^46^ was used to assess anxiety in studies 1 and 2. The Montgomery-Åsberg Depression Rating Scale^47^ was used to assess depression in study 1, while the Child Depression Rating Scale^48^ was used in study 2. The Pubertal Development Scale was used to assess pubertal developmental stage in study 2^49^.

### Structural MRI data acquisition

Brain MRI data were acquired using a Siemens Trio 3 T scanner with a 12-channel head coil (study 1) and a Siemens Prisma 3 T scanner with a 32-channel head coil (study 2). High-resolution T1-weighted images were acquired with MPRAGE (Magnetization Prepared Rapid Acquisition Gradient Echo) sequence (study 1: TR of 1900 ms, TE of 2.26 ms, and an isotropic voxel dimension of 1 mm^3^; study 2: TR of 2300 ms, TE of 2.99 ms, and an isotropic voxel dimension of 0.8 mm^3^).

### Data processing

All brain data was processed in Matlab (https://www.mathworks.com/products/matlab.html), using SPM12 (http://www.fil.ion.ucl.ac.uk/spm) and the CAT12 toolbox^50^ applying corrections for magnetic field inhomogeneities, as previously described^51,52^. All images were rigid body realigned to MNI space and resliced to 1 mm^3^ resolution. In addition, the total intracranial volume (TIV) was calculated by classifying images as gray matter (GM), white matter (WM), and cerebrospinal fluid (CSF) and adding the sub-volumes of these compartments (TIV = GM + WM + CSF).

### Callosal thickness estimation

Using the processed images, the corpus callosum was manually outlined by one rater (A.N.) in each brain’s midsagittal section^53^. The callosal traces were extracted and automatically processed in several successive steps^38,54,55^. More specifically, the callosal outlines were separated into 100 nodes and re-sampled at regular intervals rendering spatially uniform the discrete points comprising the two boundaries. Then, a new midline curve was created by calculating the 2D average from the 100 equidistant nodes representing the upper and the lower callosal boundaries. Finally, the distances between the 100 nodes of the upper as well as the lower callosal boundaries to the 100 nodes of the midline curve were calculated. These point-wise callosal distances constituted the variables of interest in the subsequent statistical analyses.

### Statistical analyses

All statistical analyses were conducted in Matlab (The MathWorks, Natick, MA) using mass-univariate general linear models to reflect the hypotheses stated in the pre-registration (https://aspredicted.org/blind.php?x=SDC_UKE). The group analysis compared control participants and participants with AN using ANCOVA, with a statistical threshold of *P* < 0.05, two-tailed. We also conducted four correlation analyses within the AN group: the first analysis examined the link between callosal thickness and BMI in all AN participants (analysis A); the second examined the link between callosal thickness and illness duration in acutely underweight and partially weight-restored AN participants (analysis B) (see Supplementary Material for subgroup definitions); the third examined the link between callosal thickness and EDE in all AN participants (analysis C); and the fourth examined the link between callosal thickness and YBC-EDS in all AN participants (analysis D). For all analyses—group comparisons and correlations—age, TIV, scanner, education, medication, and pubertal score were included as nuisance variables. For analyses C and D, BMI was additionally included as a nuisance variable. All statistical tests were two-tailed, and α was set at 0.05. To control for multiple comparisons, a Monte Carlo simulation with 10,000 permutations was employed, as previously described^56^.

## Results

### Study sample

Altogether, 120 participants from two studies—one acquired on a Siemens Trio 3T scanner (study 1), and another acquired on a Siemens Prisma 3T scanner (study 2)—comprising 60 participants with AN and 60 controls were included. Of those 120 participants, seven were excluded due to image artifacts such as ghosting or excessive motion that rendered the images unusable for further processing. Another four participants had missing values for education or illness duration and were excluded due to incomplete data. Of the 109 that survived the quality control, only seven were males (two AN and five controls). Given the low numbers, the males were excluded from the analysis to have a more homogeneous sample of 102 female participants.

Study 1 did not evaluate puberty scores. For that study, puberty scores were set to maximal for all adults. In study 2, four participants (2 AN, 2 controls) were missing puberty scores and were excluded (pubertal stage has been associated with callosal growth)^57^. This left 98 participants (48 with AN and 50 controls, all female) for inclusion in the final sample. There were 22 adolescent AN, 12 adolescent controls, 26 adult AN, and 38 adult controls. See Table 1 for demographics and psychometrics as well as missing data.

**Table 1:** Demographic and behavioural characteristics of the sample.

	AN (*n* = 48)	Control (*n* = 50*)*	Statistic	*P* value
Age (years)	18.9 ± 4.8	19.6 ± 4.1	t = 0.79	0.43
Education (years)	12.0 ± 3.8	12.6 ± 2.9	t = 0.95	0.35
Pubertal status	17.4 ± 4.03	19.04 ± 1.5	t = 2.67	0.01
BMI (adults)^a^	20.03 ± 2.2	22.2 ± 2.9	t = 3.27	0.002
BMI percentile (adolescents)^b^	25 ± 16	75.0 ± 18.2	t = 8.31	< 0.001
Duration of illness (months)	54.7 ± 52.35	NA		
Medicated/unmedicated	15/33	0/50		
EDE—total score^c^	3.1 ± 1.6	0.6 ± 0.7	t = − 9.15	< 0.001
EDE—shape concern^c^	3.9 ± 1.8	0.9 ± 1.2	t = − 8.30	< 0.001
YBC—total score^d^	23.0 ± 9.9	2.2 ± 3.7	t = − 12.33	< 0.001
HAMA	10.9 ± 7.9	4.6 ± 4.2	t = − 4.87	< 0.001
MADRS^e^	10.04 ± 8.7	1.1 ± 1.9	t = − 4.92	< 0.001
CDRS T-score^f^	60.7 ± 11.6	44.5 ± 8.5	t = − 5.50	< 0.001

^a^Data for 26 AN and 38 controls (adult).

^b^Data for 22 AN and 12 controls (adolescent).

^c^EDE was not administered to controls (n = 26) in study 1.

^d^Data missing for 5 AN.

^e^Scale was administered in study 1 (n = 24 AN and n = 26 adult controls).

^f^Scale was administered study 2 (n = 24 AN and n = 24 controls).

### Group comparisons

**Figure 1:**
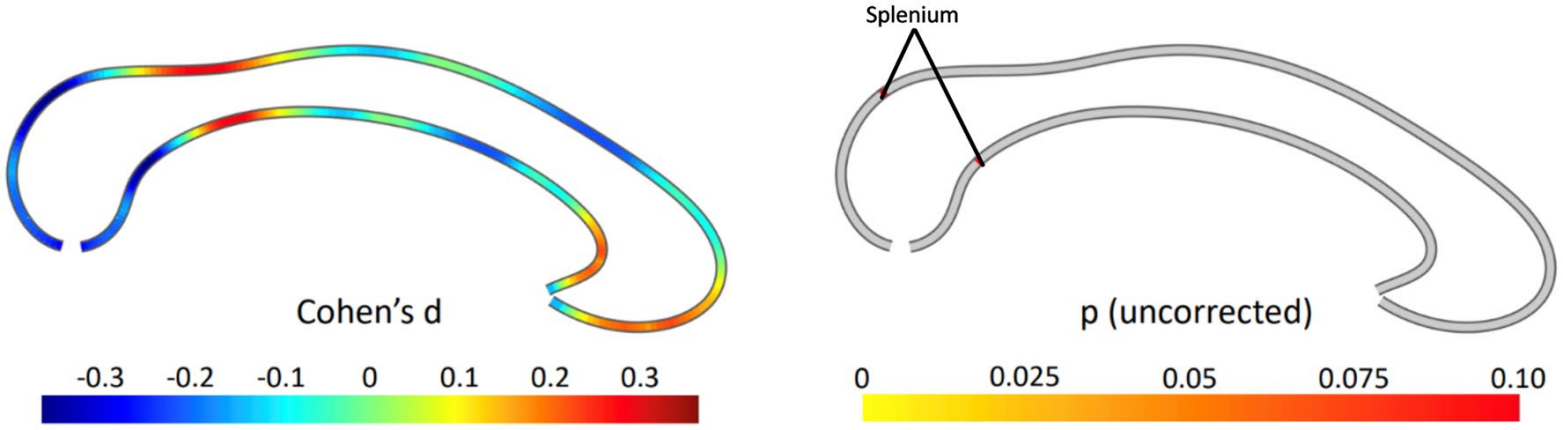
Group comparison between anorexia and controls. The effect size is shown on the left, where negative values indicate a thinner corpus callosum in anorexia compared to controls. The right panel depicts the significance (uncorrected) from a two-tailed *t*-test. All p-values are greater than 0.05 throughout, with significant areas labelled.

There were no significant differences in AN and control participants in callosal thickness (the maximum difference was t = 1.716, d.f. = 90, *P* = 0.097) (Fig. 1). We conducted an exploratory one-way ANCOVA to discern if differences were present among the subgroups of acutely underweight AN (n = 11), partially weight-restored AN (n = 8), weight-restored AN (n = 29), and healthy controls (n = 50), also controlling for age, TIV, scanner, education, medication, and pubertal status. There were no significant differences among groups (the maximum difference was *F* = 2.031, d.f. = 2.93;88, *P* = 0.209) (Fig. S1).

### Associations with BMI

**Figure 2:**
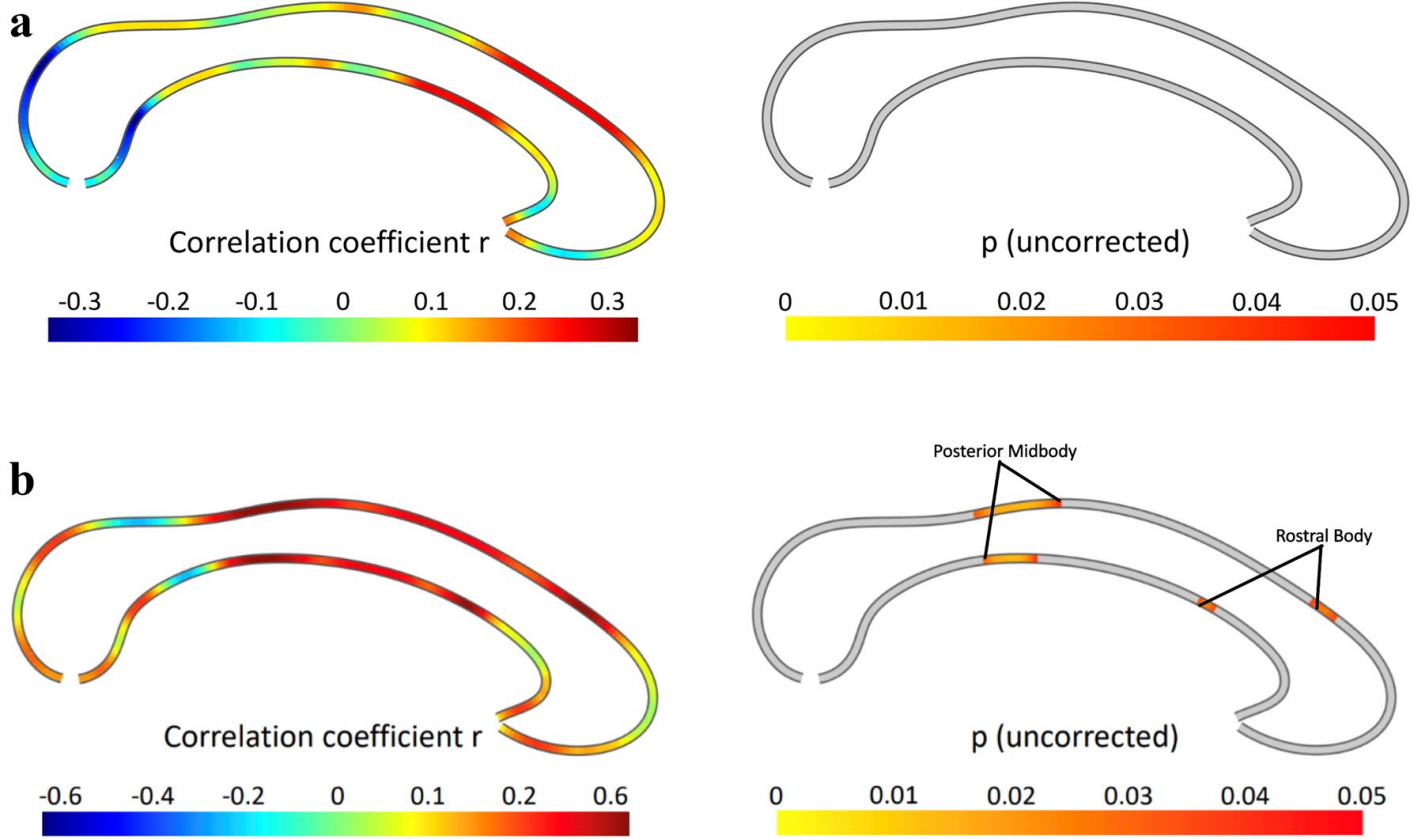
Correlations between callosal thickness and (**a**) BMI, and (**b**) illness duration. The effect size is shown on the left, where positive values indicate a positive association. The right panel depicts the significance of correlations, thresholded at p ≤ 0.05 (uncorrected). There were no significant associations.

There was no significant association between callosal thickness and BMI in the AN cohort (n = 48; the highest partial correlation was r = 0.293, *P* = 0.060) (Fig. 2a).

### Associations with illness duration

There was no significant negative association between callosal thickness and illness duration in the acutely underweight and partially weight-restored AN participants (n = 19) and only small positive associations in the rostral body and posterior midbody. However, these did not survive the correction for multiple comparisons with permutation testing (*P* = 0.109, Fig. 2b).

### Associations with clinical symptoms

#### EDE shape concern scores

**Figure 3:**
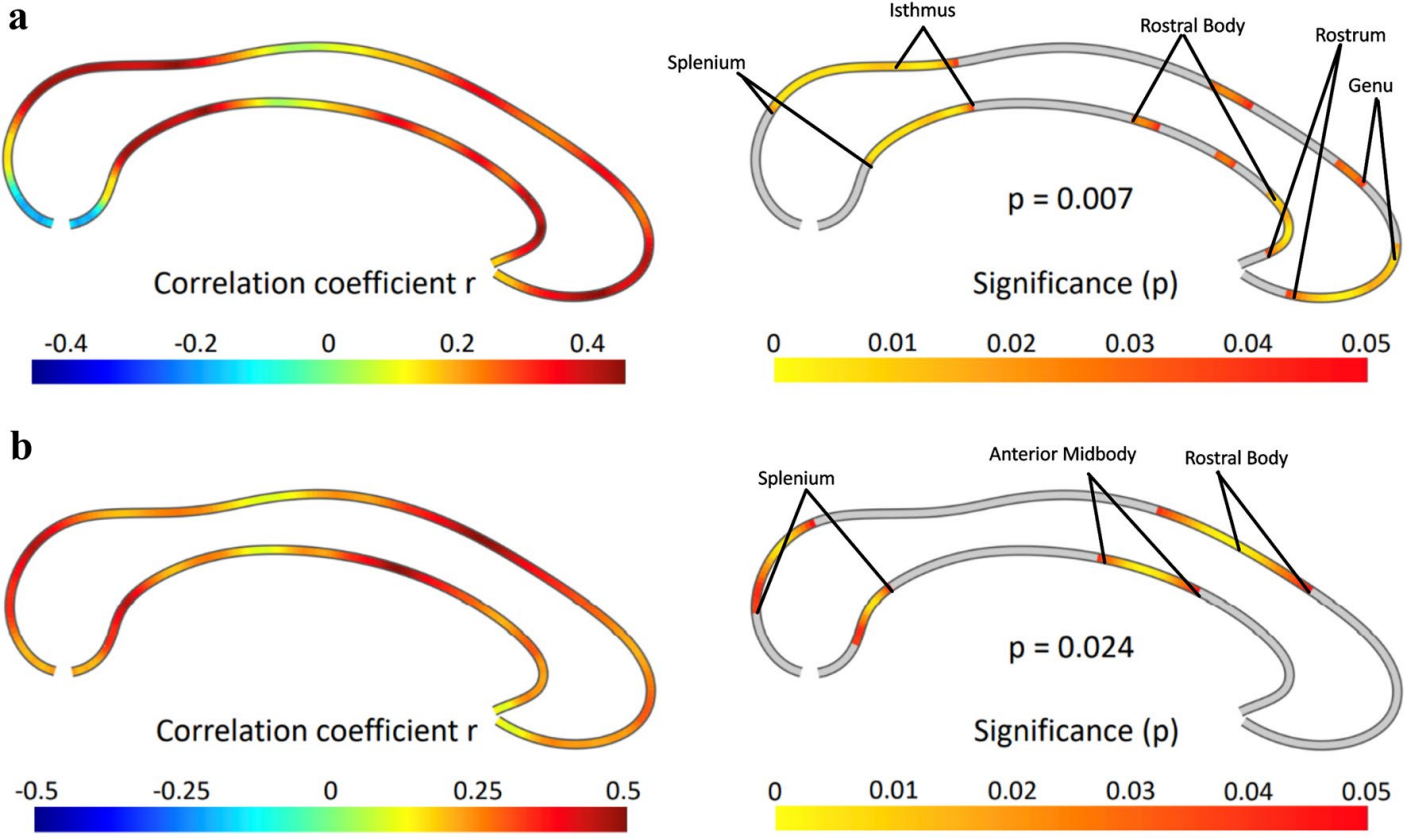
Correlations between callosal thickness and (**a**) EDE Shape Concerns, and (**b**) YBC-EDS scores. The effect size is shown on the left, where positive values indicate a positive association. The right panel depicts the significance of a positive correlation using a two-tailed test, with significant areas labelled. The results survive correction for multiple comparisons. *EDE* Eating Disorder Examination, *YBC-EDS* Yale–Brown–Cornell Eating Disorder Scale.

As hypothesized, there were significant correlations between EDE Shape Concern Scores and callosal thickness in the AN cohort (n = 48), surviving multiple comparisons correction with permutation testing (*P* = 0.007). The significant positive correlations were evident in the rostrum, genu, and rostral body, as well as the isthmus and splenium (Fig. 3a).

#### YBC scores

As hypothesized, there were significant correlations between YBC total scores and callosal thickness in the AN cohort (n = 43), surviving multiple comparisons correction with permutation testing (*P* = 0.024). The significant positive correlations were evident in the rostral body and anterior midbody as well as the splenium (Fig. 3b).

## Discussion

This is the first study to examine CC thickness in individuals with AN. The major findings included, as hypothesized, significant positive associations between CC thickness and degree of body shape concerns as well as the degree of eating disorder-related obsessive thoughts and compulsive behaviours. Contrary to our predictions, there were no significant group differences in CC thickness between those with AN and healthy controls. Further, contrary to our prediction, there were no significant associations between CC thickness and BMI in the AN sample, or between CC thickness and illness duration in the acutely underweight or partially weight-restored AN cohorts.

We observed that greater CC thickness was associated with worse body shape concerns, as indexed by the shape subscale of the EDE. Higher scores on this subscale indicate greater preoccupations with body shape, influence of body shape on one’s self evaluation, fear of gaining weight, dissatisfaction with one’s weight, discomfort with seeing one’s body, discomfort and/or avoidance of others seeing their body, feeling fat, and desires for a flat stomach^42^. In a previous meta-analysis of DTI studies, lower FA in the CC body was observed in those with AN compared with healthy controls^18^. More recent studies also found lower FA^19^, as well as lower tract volume^20^ in the CC body. Although group differences in CC thickness were not evident in the current study, similar regions of the CC body—anterior midbody and the rostral body—were associated with body shape concerns. The CC body contains fibres connecting right and left frontal hemispheres, primarily between the motor and somatosensory cortices^58^. These portions of the CC may be involved in body perception. Specifically, the anterior midbody has been shown to be involved in interpreting limb gestures^59^. Further, anterior CC callostomy patients are unable to imitate gestures of other individuals^60^. In addition, in the current study there were associations between shape concerns and thickness in the rostrum and genu, which connect right and left association cortical areas^61,62^. These may be involved in higher cognitive functions such as self-appraisals, including content related to one’s body image. Further, there were associations between body shape concerns and thickness in the isthmus and splenium, which are involved in tactile, auditory, and visual functions^58^. These regions, involved in inter-hemispheric integration of visual and visuospatial information, therefore may also be relevant for body visual perception. One speculation from these results is that greater thickness could be linked to poorer interhemispheric exchange of visual and visuospatial information, conferring more distorted perception of one’s body and leading to worse body image distortions in those with AN. However, the neural mechanisms for this are not clear. Future studies could directly investigate this by using, for example, multimodal functional and structural imaging along with direct behavioural measures of body perception.

An additional finding was that CC thickness was associated with the severity of eating disorder-related obsessive thoughts and compulsive behaviours (YBC scores). Specifically, this was observed in the splenium, anterior midbody, and the rostral body. A study of individuals with OCD found inverse associations between severity of compulsions with total CC area, as well as associations specifically with the area of the isthmus and splenium^37^. Thus, similar posterior CC regions found to be associated with obsessions and compulsions in OCD were also found to be associated with obsessions and compulsions in the current AN study. However, the direction of the relationships differs; in OCD there were negative associations while in AN there were positive associations. The reasons for the different directions of these associations are unclear, and direct comparisons between the current AN study and the previous OCD study are hindered by the fact that the previous OCD study measured CC area rather than thickness. Nevertheless, there may be overlapping associations in OCD and AN linking posterior CC morphology and obsessive–compulsive symptomatology. As the posterior CC connects occipital, superior temporal, and posterior parietal regions^63,64^, future research may explore links between visuospatial functioning, interhemispheric structural (and functional) connections, and obsessive–compulsive symptoms trans diagnostically across OCD and AN.

There were no significant associations between CC thickness and either BMI or illness duration, contrary to our predictions. The lack of significant associations with BMI suggests the possibility that low-weight states, reflecting the current degree of starvation and possibly nutritional status, may not be associated with CC thickness in these cohorts. Further, the lack of a significant association with illness duration could point to the absence of a cumulative impact of illness effects on white matter thickness in the CC. The typical illness course in those with AN consists of an acute underweight starvation state followed by either partial or full weight restoration. The partially weight-restored state, and even the full weight-restored state^65^, might include an attenuated state of reduced caloric intake or poor nutrition. Thus, “scar” effects of acute starvation and malnutrition, periods of persistent poor nutrition even in partially- or fully weight-restored states, and/or other chronic illness effects in AN such as those related to diminished quality of life and functionality, may not be manifested in this aspect of CC morphology. Thus, the interesting observation that subjective clinical variables (EDE and YBC-EDS) but not the objective variables (illness duration and BMI) were associated with CC thickness could be due to regional CC thickness mediating persistent aberrant thoughts and perceptions related to body, food, and weight, as well as repetitive behaviours, but not potential “scar” or malnutrition effects that illness duration and BMI may reflect. However, longitudinal studies are necessary to definitively elucidate the effects of these chronic illness elements. 

There are several limitations to consider. As this was a cross-sectional study, cause-and-effect inferences regarding the associations between clinical symptoms and CC thickness cannot be ascertained. It is not known, for example, if pre-illness CC thickness patterns (and any patterns of interhemispheric neural communications, e.g. that these may be linked to) contribute to risk for AN symptoms. Alternatively, the symptoms themselves, or compensatory or other secondary effects of the symptoms, may have affected regional CC thickness to create these patterns of associations with clinical symptoms. Another limitation is the modest sample size, which may have affected the ability to detect group differences and, particularly as multiple covariates were necessary, affected the stability of the correlation estimates. Further, the small subsamples of individuals in the underweight, partially weight-restored, and weight-restored groups, in addition to adolescents and adults, limited the power for the sub-analyses. Further, the fact that this was a heterogeneous sample of adults and adolescents, medicated and unmedicated, obtained across two separate studies, may have affected the results. We addressed this by covarying for age, TIV, scanner, education, medication, and pubertal score across all analyses; nevertheless, there may have been other sources of variance of non-interest that were not accounted for. 

In terms of clinical implications, while CC thickness may not represent a biomarker specifically of the diagnostic category of AN, the degree of thickness may reflect white matter structural relationships with two important phenotypes: body shape concerns and eating disorder-related obsessions and compulsions. As cause-and-effect relationships cannot be determined, future investigations of cohorts followed prospectively could focus on CC thickness to determine if pre-illness patterns may predict future symptoms and thus contribute to a risk profile for the development of AN. Future transdiagnostic studies may be useful as well, since body image disturbances and obsessions and compulsions occur not only in other eating disorders such as bulimia nervosa, but also in body dysmorphic disorder^1,66^. Transdiagnostic studies may also extend to other obsessive–compulsive and related disorders such as OCD, since, as mentioned above, CC thickness relationships with obsessive–compulsive symptoms have been found^36,37^. Finally, findings from this study could spur studies to examine multimodal interhemispheric regional functional and structural connectivity; this would be especially informative if done in the context of experimental paradigms probing body perception and habit or ritual formation.

In conclusion, this study revealed regional CC thickness patterns in individuals with AN that are associated with important clinical phenotypes. There were no significant differences, however, in CC thickness between AN and healthy controls, nor links with BMI or illness duration within the AN group. Specifically, thicker CC in the anterior, body, and posterior regions were tied to more pronounced body image disturbances, possibly reflecting impaired interhemispheric communication affecting body perception. Additionally, CC thickness in the body and posterior regions were linked to the severity of obsessive–compulsive symptoms, suggesting shared neural patterns with OCD. These findings offer insights into structural associations with critical symptom dimensions in AN and may have additional implications for future mechanistic and transdiagnostic investigations.

### Supplementary Information

Supplementary Information.

## Data Availability

Data for study 2 is available through the National Institute of Mental Health Data Archive (https://nda.nih.gov/edit_collection.html?id=2565). The raw data for Study 1 supporting the conclusions of this article will be made available by the authors upon request.
