# Supplementary material for: Corpus callosum morphology and relationships to illness phenotypes in individuals with anorexia nervosa
Source: Sci Rep. 2024 May 15;14:11112. doi: 10.1038/s41598-024-61841-6 (PMC11096409; doi:10.1038/s41598-024-61841-6)
Supplement: Supplementary file 1 — Supplementary Information. [file 41598_2024_61841_MOESM1_ESM.docx]

**Supplementary Materials**

A. Supplementary Methods – Fig. S1

B. Supplementary Results – Fig. S2-S7

C. References

1. **Supplementary Methods:**

*Study Criteria*

We defined the subcategories of anorexia nervosa participants similar to BMI thresholds that the ENIGMA Eating Disorder Consortium https://enigma.ini.usc.edu/wp-content/uploads/2019/02/grouping_guidelines_ENIGMA_AN_ms_201812.pdf has previously outlined.

(Also see studies published using similar criteria (Gupta et al. 2022; Walton et al. 2022).)

- *Acutely underweight:*  BMI of < 17.5 or < 10^th^ age-adjusted BMI percentile
- *partially weight-restored:* BMI is ≥ 17.5 or ≥10^th^ percentile but < 18.5 or < 25^th^ percentile
- *weight restored:* BMI is ≥ 18.5 or ≥ 25^th^ percentile

Fig S1. CONSORT flow diagram of the cross-sectional study


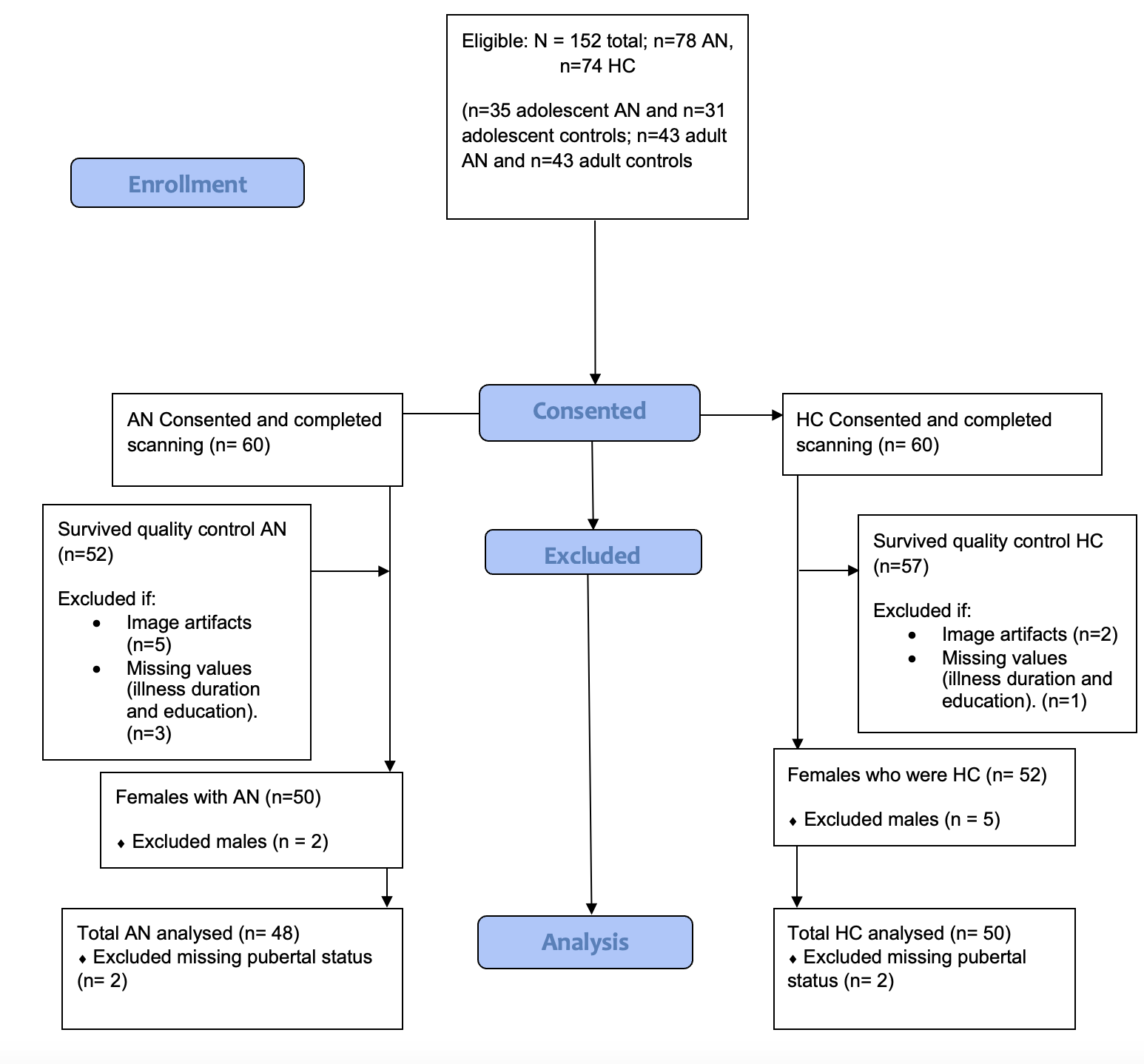


1. **Supplementary Results:**

Fig. S2. Callosal thickness in the subgroups.


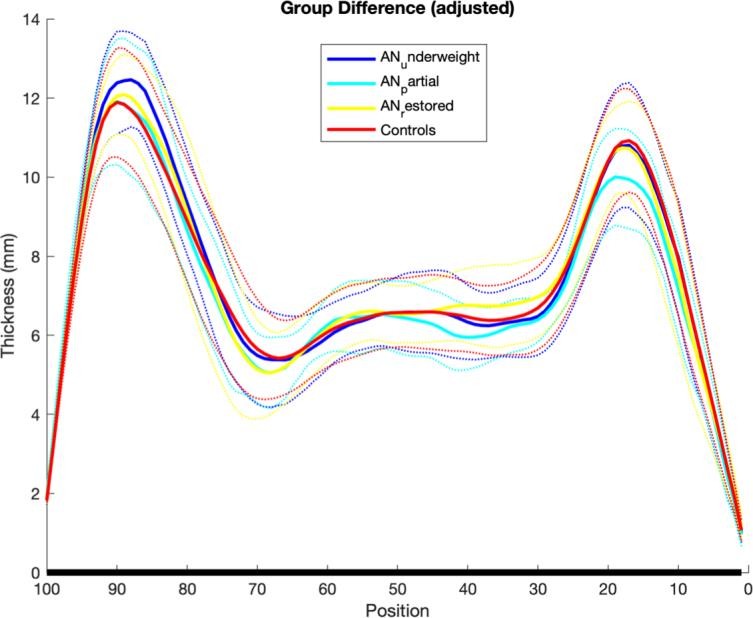


**Figure S2:** Means (solid lines) and standard deviations (dotted lines) for thickness are plotted for each group from the tip of the rostrum (right) to the bottom of the splenium (left). Thickness values are adjusted for age, TIV, scanner, education, medication, and pubertal score. There were no significant differences among groups. The maximum difference was *F*=2.031, d.f.=2.93;88, *P*=0.209, uncorrected.

Fig. S3. Callosal thickness with only three subgroups (underweight AN + partially weight-restored vs weight restored vs HC).


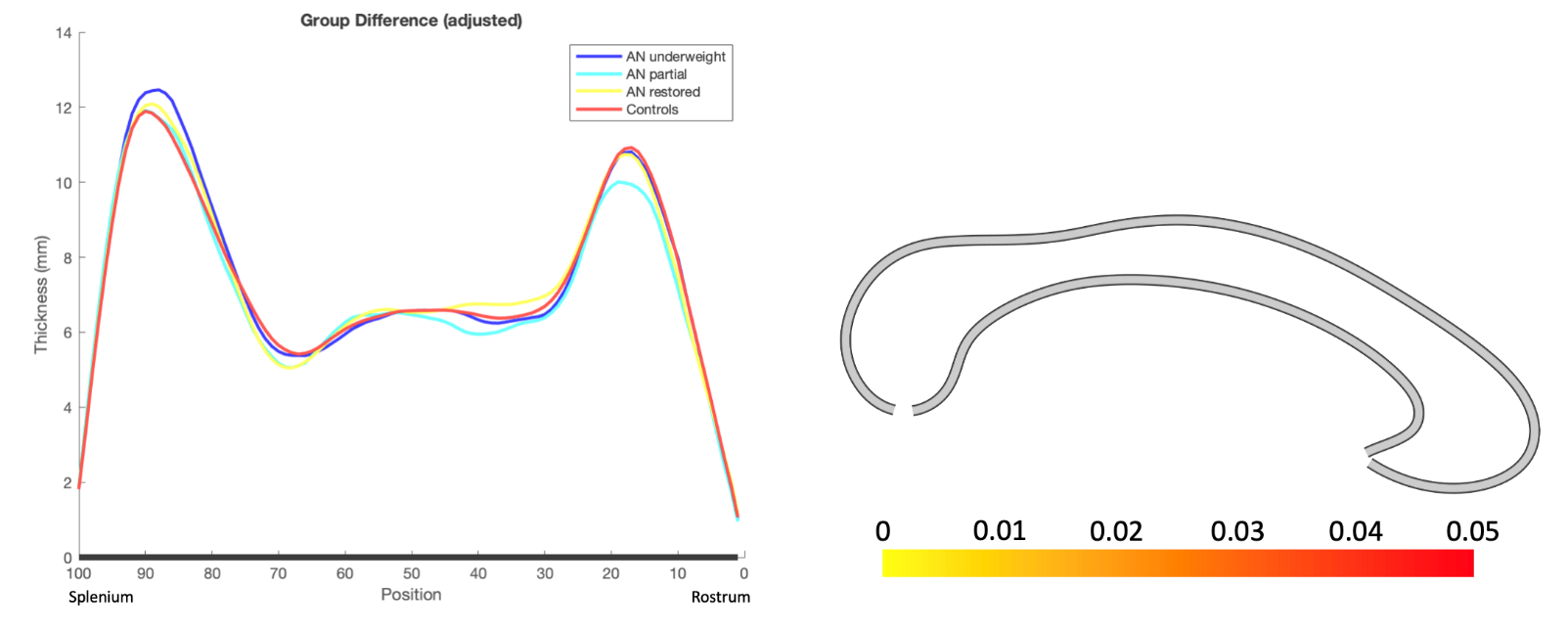


**Figure S3:** On the left are the results for the point-wise callosal thickness plotted for each subgroup. Means (solid lines) for thickness are plotted for each group from the tip of the rostrum (right) to the bottom of the splenium (left). Thickness values are adjusted for age, TIV, scanner, education, medication, and pubertal score. The puberty score is estimated to be “adult” for the cohort that did not include adolescents and therefore did not get the puberty scores. There were no significant differences among groups.

Fig. S4. Correlation between MADRS and callosal thickness in the anorexia population


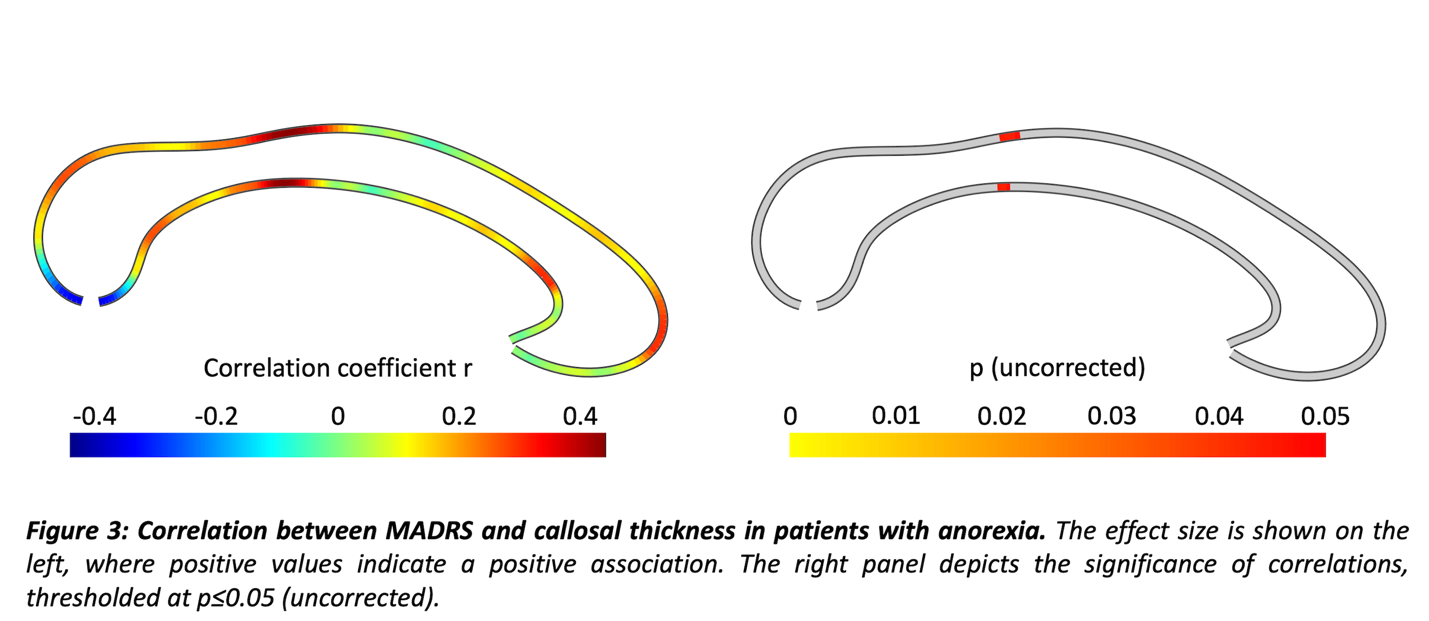
**Figure 4S.** The effect size is shown on the left, where positive values indicate a positive association. The right panel depicts the significance of correlations, thresholded at p ≤ 0.05 (uncorrected).

Fig S5. Correlation between CDRS and callosal thickness in the anorexia population


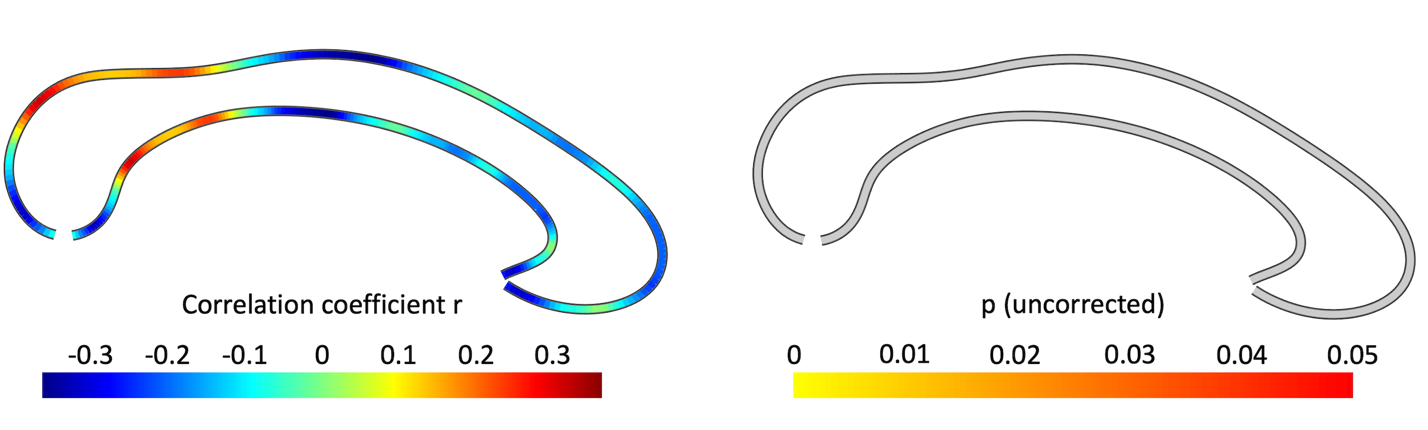
**Figure 5S.** The effect size is shown on the left, where positive values indicate a positive association. As depicted in the right panel, significance did not reach p ≤ 0.05 (uncorrected) anywhere along the corpus callosum.

Fig S6. Correlation between HAMA and callosal thickness in the anorexia population


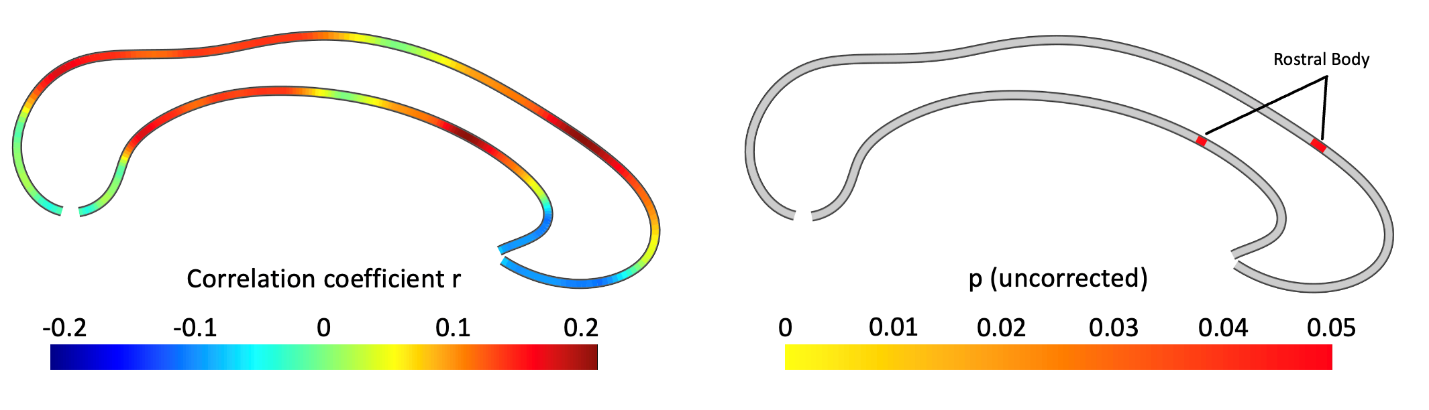
**Figure 6S**. The effect size is shown on the left, where positive values indicate a positive association. The right panel depicts the significance of correlations, thresholded at p≤0.05 (uncorrected).

Fig S7. Correlation between duration of illness and callosal thickness in the anorexia population


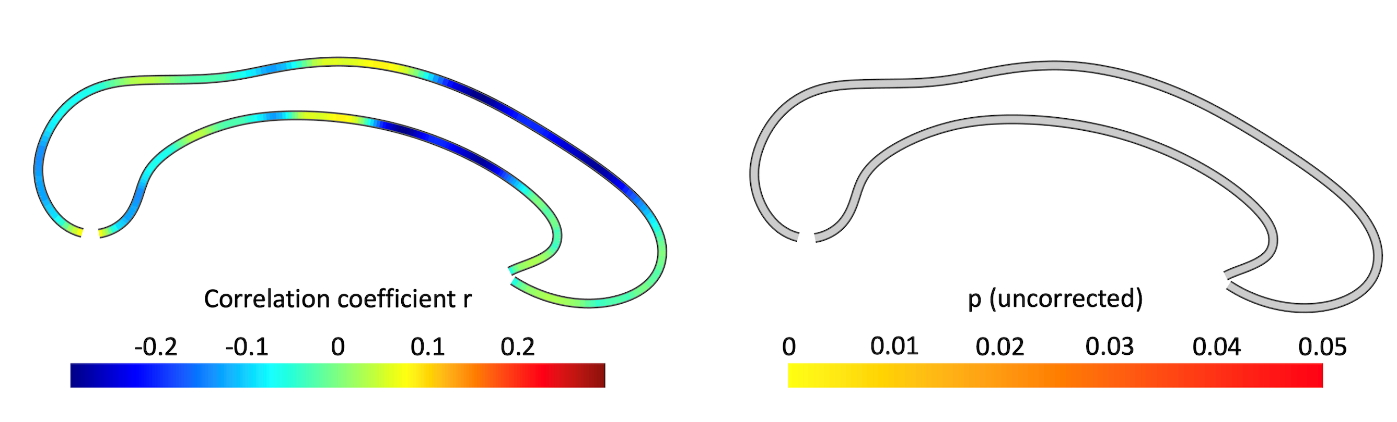


**Figure 7S**. The effect size is shown on the left, where positive values indicate a positive association. The right panel depicts the significance of correlations, thresholded at p≤0.05 (uncorrected).

1. **References:**

- Gupta A, Bhatt RR, Rivera-Cancel A, Makkar R, Kragel, PA, Rodriguez T, Graner JL, *et al*. (2022): Complex Functional Brain Network Properties in Anorexia Nervosa. *Journal of Eating Disorders* 10(1): 13.
- Walton E, Bernardoni F, Batury VL, Bahnsen K, Larivière S, Abbate-Daga G, Andres-Perpiña S, *et al*. (2022): Brain Structure in Acutely Underweight and Partially Weight-Restored Individuals with Anorexia Nervosa: A Coordinated Analysis by the ENIGMA Eating Disorders Working Group. *Biological Psychiatry* 92(9): 730–38.
